# Medical Items and News of Interest to the Physician

**Published:** 1883-04

**Authors:** 


### For the Use of Physicians.

CULLED FROM THE STANDARD MEDICAL JOURNALS OF THE WORLD.

NOTE.—These formulæ are intended only for the regular practitioner of medicine, and should be employed only by him, or under his immediate supervision.

**Cough Medicine.**

℞

Syrupi Doverinæ.
Syrupi senegæ.
Syrupi scillæ, aa f. ℥ j. M.

Dose, a teaspoonful four or five times a day.

**Odor of the Lochia.**

℞

Acid. carbolici glacial, ℥ j.

Glycerini, ℥ j.

Aquæ puræ, ℥ viij. M.

Sig. A tablespoonful in eight ounces of warm water twice a day as a vaginal injection.

**Spasm of the Glottis.**

Dr. Joseph Williams, of Boston, caused almost immediate arrest of stridulous respiration in an infant suffering from glottic spasm by administering, slowly, ten minims of amyl nitrite.—*Canad. Med. and Surg. Journ.*

**Psoriasis Inveterata.**

Dr. Reiter writes to Squibb's *Ephemeris*, giving an account of a cure in his own case of chronic hereditary psoriasis by the use of the tincture of burdock (Rappa major) seed. The maximum dose used was ʒ iv. t. i. d., to be kept up for four months if necessary. The doctor had suffered from the disease for many years.

**Rheumatism.**

℞

Acidi Salycilici, ʒ ij,

Potassæ Bicarb., ʒ iij.

Aquæ, ℥ ij. M.

This is an excellent remedy in rheumatism, given in teaspoonful doses every two or three hours.

**After Pains.**

℞

Pulv. g. camphor.

Cretæ pp.

Pulv. glycyrrh., aa ℈ j.

Morphiæ sulph., gr. j. M.

Sig. Ten grains, repeated if necessary in four or five hours.

**Neuralgia.**

Professor Roberts Bartholow recommends equal parts of chloroform, camphor, and hydrate of chloral as an efficient local application to allay the pain of neuralgia. This simple mixture, he recently stated to his class, is very rapid in its anodyne action on the part to which it is applied.

**Frontal Headache.**

Dr. Haley, *Australian Medical Journal*, claims that minimum doses of iodide of potassium are of great service in frontal headache. A two-grain dose dissolved in half a wineglass of water will often cure a dull headache which is situated over the eyebrow. The action of the drug is quite rapid.

**Leucorrhœa.**

℞

Zinci oxidi vel bis. carb., grs. lxxx.
Ext. belladonna, grs. xl.
Oleo theobroma, ℥ j.
Olei olive, ʒ iij. M.

Divide into eight pessaries.

Sig. One to be used every night.—*Medical Digest.*

**Ulcer in Ear.**

Dr. Miller recommends the following:

℞

Ext. eucalypt.
Aquæ, aa f. ℥ j. M.

Sig. Fill up the external meatus with the preparation two or three times a day, after cleansing the ear first with tepid water.—*Med. f Brie.*

**Nervous Cough.**

℞

Acid hydrocyanic dil., gtt. xvj.

Tinct. sanguinara, ʒ iv.

Syrup senega.

Syrup tolu, aa ʒ vj.

Aqua lauro cerasi, qs. ad. ℥ iij. M.

Sig. One or two teaspoonfuls according to age, every three or four hours.—*Bartholow.*

**Salicylic Ointment.**

℞

Acidi Salycilici, ʒ ij.

Rect. Spts., ʒ ij.

Unguent. petrolii., ℥ iij. M.

Thoroughly incorporate. It is stimulant and antiseptic, consequently is valuable in all wounds where cicatrization is tedious, or where stimulation or disinfection may be required.

**Ring Worm.**

℞

Adipis, vi ʒ.

Glycerinæ, ij ʒ.

Sodii carbonat, i ʒ.

Calcis pulv., ss ʒ.

Carbonis (lig.) pulv., jss ℥. M.

Sig. Before applying this salve remove scabs by using starch poultices. Treatment should be kept up two or three months in cases of tinea tonsurans.—*N. O. Medical Journal.*

**Piles.**

℞

Iodoform, ʒ j.

Balsam of peru, ʒ ij.

Cocoa butter,

White wax, aa ℥ iss.

Calcined magnesia, ʒ j.

Incorporate the mass thoroughly and divide into twelve suppositories. Insert one after each evacuation of the bowels and oftener if needed.—*Lou. Medical News.*

**Incontinence of Urine.**

For incontinence of urine in children, Dr. Janeway (*Medical Record*) recommends a combination of ergot, belladonna and iodide of iron.

℞

Tinct. ergot, ʒ ij.

Tinct. belladonna, ʒ j.

Syr. iodide iron, ℥ j.

Simple elixir, ℥ j. M.

One teaspoonful morning, noon and bedtime to a child ten years old.—*Medical Summary.*

**Bronchitis.**

M. Melsens having observed the good effects of the atmosphere of a stable on those suffering from pulmonary diseases, which are rightly attributed to the emanations of carbonate of ammonia, he thought that continued, yet moderate, respiration of this salt might be useful in other affections of the respiratory organs. After a serious attack of bronchitis, he decided on trying on himself the effects of carrying a little bag round his neck containing little pieces of carbonate of ammonia. From the first day the amelioration was felt, and the cough soon disappeared entirely, while often persons who suffered from chronic bronchitis also obtained relief. The use of little bags of carbonate of ammonia are intended to produce the same result as the air of a stable or a gasworks.—*Medical Press.*

**Inflamed Conjunctiva.**

A correspondent of the *Louisville Medical News*, describing a visit to the Manhattan Eye and Ear Hospital, New York, supplies the formula of a solution in very common use there for inflamed conjunctiva. It is used with an atomizer in the form of spray:

℞

Tannin, grs. x.

Soda bicarb., grs. xx.

Glycerine, ʒ ij.

Aquæ, O ij.

**Neuralgia.**

The following prescription is recommended by J. Ashburton Thompson, in neuralgia:

℞

Phosphori, gr. j.

Alcohol, ʒ v.

Glycerini, ℥ iss.

Spts. vin., recticfiat., ʒ ij,

Spts. menth. pip., ʒ ss.

Dissolve the phosphorous in the alcohol by the aid of heat; warm together the glycerine and wine, mix while hot and add the peppermint on cooling.—*Medical Gazette.*

**Malignant Pustule.**

Dr. Poladura, in La Revista de Medicina y Cirugia Praticas, relates the following case of malignant pustule, apparently cured by the hypodermic use of iodine. The patient was a shepherd, aged forty-one, of sanguine temperament and good constitution. Five days before he was seen, he presented the local and constitutional signs of malignant pustule of the face. The treatment consisted in incisions, excisions, the actual cautery, and antiseptic and emollient applications. During the next two days following, the œdema and induration had attained such a degree as to constitute a veritable monstrosity; the temperature was 40° C., and there was vomiting and great prostration. Tincture of iodine was given internally and antiseptic applications continued. Ten hypodermic injections of a two per cent. solution of tincture of iodine were administered around the zone of induration. Subsequently the symptoms became aggravated. Injections were again practiced at various spots in the œdematous region. The next day the delirium, as well as the sweats and chills, had disappeared, and the pulse was stronger. An inflammatory circle was visible at the margin of the eschar. Treatment being continued, a cure resulted—*Rev. de Ciencias Med*

**Chronic Dysentery.**

Koroniko, from *veronica parviflora*, is reported by Dr. J. Jardine ("*Chinese Customs Rep.*") as a potent remedy in cases of chronic dysentery, varying in duration from six weeks to four years, and in which from twenty to thirty motions containing blood and mucus were voided daily. Fifteen doses of tincture of koroniko reduced them to three or four daily, and a third like quantity effected a complete cure. Koroniko seems to be the vulgar name adopted in New Zealand for the plant.—*Medical Record.*

**Vesical Catarrh.**

Dr. Ciancosi claims excellent results in the treatment of vesical catarrh, by bromide of potassium in doses of one-half to three grammes daily. He explains its effect primarily by its depressing action on the vaso-motor nerves and the cardiac plexus, producing a general ischæmia of the entire organism. In addition, a local action is exerted on the vesical mucous membrane from the presence of the salt in the urine. It has been found in this fluid ten minutes after its administration by the mouth. —*Medical Record.*

**Cutting Short Acute Tonsillitis.**

Dr. Morell McKenzie says that guaiac given early will rarely fail to cut short an acute tonsillitis. The formula is as follows:

℞
Resin guaiac, ℥ ij.
Gum tragacanth, ℥ j.
Sacchar. alb., ℥ ss.
Black currant paste, q. s. M.
Divide into cccl. troches.

Sig. One every two hours.

One can also give aconite, as recommended by Ringer. If the disease is not checked, give small pellets of ice.

**Whooping Cough.**

M. Dujardin recommends the combination of the bromides and chloral as being very useful in whooping cough. He gives one dessertspoonful of the mixture in a glass of milk, to which the yolk of an egg has been added, evening and morning.

℞

Potassii bromidi, ʒ ss.

Sodii bromidi, ʒ j.

Ammonii bromidi, ʒ ss.

Syr. chloral, ℥ iss.

Aquæ, ℥ ij.

—*Medical Summary.*

**"Loomis Tonic."**

Professor A. L. Loomis, M. D., is credited with a tonic combination, which is largely used in Bellevue and Charity Hospitals, New York, under the name "Loomis' Tonic," and which is prepared after the following formula:

℞

Quiniæ sulphatis, gr. xxx.

Acidi sulph. dil., q. s.

Tinct. ferri chlor, fl ℥ ss.

Sptr. chloroformi, fl ʒ vj.

Aquæ, fl ℥ ij.

Glycerinæ, q. s. ad. fl ℥ iv. M.

Dose—a teaspoonful.

**Diphtheria.**

Dr. Lamorre, St. Germain, has lately employed petroleum oil topically, with very encouraging results, in an epidemic of diphtheria. Archambault has tried it in the Children's Hospital, but without any obvious advantage. In two severe cases it was used with the result that one died and the other recovered. In the case which recovered there were albuminuria.

Petroleum is a rapid solvent of the false membranes, and must possess distinct advantages under such circumstances. This fact, coupled with its antiseptic property, renders it a promising remedy, although its odor always renders its use disagreeable.—*Detroit Clinic.*

**Epilepsy.**

When I commenced practice, in 1833, nitrate of silver was the grand remedy for this complaint. After repeated failures with it, I was told by Dr. Boyd, an octogenarian of our city, that he had no trouble in its cure. He had treated a man successfully, who had not earned a dollar in twenty years, who afterwards supported his family. I gladly adopted his practice, and have been successful ever since. The remedy, oxide of zinc. "Begin with one-half grain dose, three times a day for twenty-four doses. Then one grain for twenty-four doses. Then one and one-half grains, and so on up to fifteen grains three times a day, rubbing the spine with stramonium ointment, morning and evening, and other stimulating embrocations," which I have seen used. Since then I have been successful; never going beyond ten grains, except in one case, of a hard drinker and opium taker, who at the time I commenced with him had been treated for a year with bromide of potash, impairing his memory badly, which was restored by the use of zinc.—*E. Vanderpoel, M. D., in Medical and Surgical Reporter.*

**Erysipelas.**

Dr. Rothe recommends (*Memorabilien*) the application of the following liquid every two hours to the affected parts:

℞

Acid. carbolic.

Alcohol, āā 1 part.

Ol. tereb inthinæ, 2 parts.

Tr. iodinii, 1 part.

Glycerinæ, 5 parts. M.

This mixture causes no pain. Internally, he recommends quinine and digitalis, and an emetic if indicated.—*Med. and Surg. Reporter*.

---

**Disguising the Taste of Quinine.**

J. L. Lilly, in the *Indiana Pharmacist*, says that the following syrup has proved to be very satisfactory in disguising the taste of quinine:

℞

Fluid extract yerba santa, 4 parts.
Water, 8 parts.
Powd. pumice, 1 part.
Granulated sugar, 14 parts.

Mix the fluid extract with the water, evaporate to seven parts, shake with pumice, allow to stand, decant, add sufficient water to preserve measure, then with heat dissolve sugar.

---

**Cancer of the Uterus.**

A few years ago Prof. John Clay, of Manchester, England, published an account of a number of cases treated by him successfully by means of Chian turpentine. The article was published in 1880 in the *London Lancet*, and more in subsequent numbers of that journal. The cases embraced all kinds of carcinoma uteri, involving the os, cervix and body of the organ. The turpentine was sometimes given in the pillular form, sometimes in mixture. Six grains of Chian turpentine were given, in combination with four grains of sulphur, made into two pills, every fourth hour; or an etherial solution made by adding one part of the turpentine to two of sulphuric ether, and this added to a solution of gum tragacanth and sulphur, given three times a day, formed an elegant mixture. One or the other of these preparations, and sometimes substituting one for the other, formed the chief treatment of his cases. Both scirrhous and epithelial cancer readily yielded to this remedy. Coming from such a source, the Chian turpentine has been extensively tried in Europe and in this country in the treatment of cancer uteri, but not with the success claimed for it by Mr. C. Mr. C. accounts for the failure of the drug in other hands to the employment of a spurious article. However, observers are not wanting who failed to obtain benefit in cancer uteri from Chian turpentine, obtained from samples pronounced pure by Mr. Clay himself.

---

**Diphtheria.**

At a meeting of the Baltimore Medical Association, held October 23, 1882, Dr. C. H. Jones reported several cases of malignant diphtheria, after which Dr. Erich said that if diphtheria were seen in twenty-four hours from the beginning not one case in a hundred need die. When it affects the larynx and trachea there might not be time to get the results. Dr. Erich's treatment (which has already been published in a previous number of this journal) consists in the administration of the following formulæ:

℞

Tinct. ferri chloridi, ʒ ij.
Quiniæ sulph., gr. viij.
Syrup simp., ℥ iv.

Sig. One teaspoonful every half to one hour, day and night, and no water to be taken immediately after.

℞

Acid. benzoic., ʒ ij.

Sodii benzoat., ʒ iij.

Aquæ, ℥ xij.

Sig. Teaspoonful every hour. These are to be alternated. The last should be dropped as the patient improves, as it is liable to disagree with the stomach. Usually by these means the fauces are cleaned off within the first twenty-four hours, leaving bare the rough surface where the deposit had been.

---

**Infantile Diarrhœa.**

Christopher Eliot, M. D., writes, in *The Practitioner* of December, 1882, that he now seldom employs any other remedy than infusion of chamomile (*Anthemis nobilis*) in infantile diarrhœa. It is especially useful for the diarrhœa occurring during dentition, when the stools are many in number, green or slimy, and streaked with blood. Pain or cramp especially indicate its use, and a few doses will quickly calm a fretful child. ℥ ss. to ℥ i. of the infusion may be given to a child under one year of age or double that quantity to a child over that age, and it may be repeated thrice or oftener daily, according to the severity of the case.

**Cough Mixture.**

The following formula, said to have originated with the late Prof. Pancoast, of Philadelphia, has the advantage of containing no opium or morphine, since many persons can not take either of these remedies without discomfort:

Wild cherry bark,

Senega, āā ʒ iv.

Ipecacuanha, ʒ ij.

Extract of conium, gr. xv.

Water q. s. ft. (by displacement) fl. ℥ viij.

Then add

Gin, ℥ i.
Compound tinct. of cardamom, ℥ i.

Two teaspoonfuls in water constitute the usual dose to relieve cough.—*Med. Bulletin.*

---

**Infantile Diarrhœa.**

Dr. Guerin, in referring to a recent communication to the *Academie de Medicine*, made by Bouchardat, remarks that for a long time he has been in the habit of combating infantile diarrhœa by mixing the milk in the sucking bottle with charcoal powder. He usually adds half a teaspoonful of the powder to one bottle of milk. The infants take the milk readily, and in a few days the greenish stools of the little patients change to a dark yellow, while their consistence becomes increased. In addition to the admixture of powdered charcoal, the milk is diluted by one-half or one-third of its bulk of sugared water. He has frequently seen intractable summer complaints yield in a few days to this treatment.—*South. Pract.*

---

**Vomiting.**

The use of the sulpho-carbolate of sodium in flatulent dyspepsia is well known as a remedy for the vomiting of pregnancy. I have used it in this affection for several years, and find it rarely fails to give some relief. In some cases the benefit is extremely marked. I give it in doses of seven grains in half an ounce of water. Though sometimes decidedly useful in the vomiting of displaced or other abnormal conditions of the uterus, it is less uniformly so than in pregnancy, probably because flatulence is a less constant factor in the former cases. Where deep nerve disturbance exists, we must trust to more powerful remedies, hypodermic morphia or atropine, or surgical procedures. The drug will, perhaps, be useful against sea-sickness, taken every two hours from the time of sailing. In one case—the only one tried—it appeared to have good effect.—Philip Miall, in *British Med. Jour.*

**Neuralgia.**

Dr. McColganan extols the value of the ether or rhigolene spray for the instantaneous relief principally of facial neuralgia. He first had occasion to observe its good effects upon his own person, he having suffered greatly from facial neuralgia. Since curing himself he has had occasion to test its efficacy in about twenty cases. The result was invariably a most gratifying success. In many instances a permanent cure was established. He attempts to explain its action by supposing a complete change to take place in the nutrition of the affected nerve in consequence of the intense cold acting as a revulsive.—*Southern Practitioner.*

---

**Venereal and Common Warts.**

Prof. Unna recommends for the treatment both of venereal and of ordinary warts the continuous application of unguentum hydrargyri, containing 5 per cent. of arsenic. In the case of a young girl upon whose hands were a hundred or more warts, the application for three weeks of a plaster, containing in each 0.2 square meter 10 grammes of arsenic and five grammes of mercury, caused entire disappearance of the disease without any irritation of the healthy skin. Cure was effected not by necrotic destruction of the warts, such as occurs in the use of caustics, but by resorption, as in cases of spontaneous cure.—*Monatschrifte fur prakt. Derm.*

---

**Excessive Sweating.**

Dr. T. H. Currie, Lebanon, N. H., says in *Michigan Medical News*:

For over thirty years I have used the following prescription, without a single failure, in sweats from whatever cause:

Alcohol, O j.
Sulphate of quinine, ʒ j. M.

Wet a small sponge with it and bathe the body and limbs, a small surface at a time, care being taken not to expose the body to a draught of air in doing it. In one case a neighboring physician was poisoned while dressing a mortified finger. He suffered untold misery, and was drenched with perspiration for a number of days, and his life despaired of. When I saw him I ordered him to be bathed immediately in the above solution, and that this be repeated once in two hours. The third application stopped all perspiration, and convalescence began at once.—*Quinologist.*

**Cold.**

The following is recommended by M. Vigier as a pectoral in place of the nostrums so commonly used:

℞

Syrup adianti (s. capillaire), 200 grm.
Ext. opii, 0.10 grm.
Ext. hyoscyami, 0.20 grm.
Ext. aconiti (aq.), 0.30 grm.

Dissolve the extracts in distilled water (about 3 or 4 grms.), filter, and add the syrup. This is to be taken in tablespoonful doses three or four times daily.—*La France Med.* [Probably the aqueous and not the alcoholic extract of aconite is meant.]—*Ed. Med. Times, Philadelphia.*

---

**Vaginitis.**

M. Terrillon proposes a method of treatment which consists essentially in the introduction into the vagina of the following ointment:

℞

Ac. tannic, 50 grms.
Amyli, 150 grms.
Ung. petrolei, 150 grms. M.

This ointment is placed in a sort of speculum, so arranged that the ointment can be forced out as the instrument is withdrawn from the vagina. A small tampon of cotton may be introduced. Generally from fifteen to twenty grams of the unguent is sufficient at one application, and it need not be repeated for seven or eight days.

---

**Hydrobromic Acid.**

Dr. W. B. Moir says, in the *Lancet*, that he has obtained some excellent results from the use of this acid. A young lady complaining of severe and frequent headaches, accompained with flushing of the face, and at times with ringing in the ears, was encountered. She seemed perfectly healthy, and there was no appreciable cause for this condition. She was ordered fifteen minims of the acid, thrice daily, after meals, in a little sweetened water. This treatment was continued for three weeks (the dose being increased first to twenty and subsequently to twenty-five minims). The happiest possible results ensued, and complete relief was afforded. Given in combination with quinine, he has found it to mitigate or entirely prevent the headache which often accompanies the use of that drug. In a case of persistent toothache, occurring during, pregnancy, he obtained very satisfactory results from the use of this acid.—*Med. and Surg. Reporter.*

**Constipation.**

Where constipation is due to torpor of the muscular layer of the intestine, combined with defectiye secretion of the mucous membrane, Dr. Bartholow uses either one of these formulæ:

℞

Tc. nucis vomicæ.
Tc. belladonnæ.
Tc. physostigmæ., aa f. ℥ ij. M.

Sig. Thirty drops in water, morning and evening, or,

℞

Ex. physostigmæ.
Ex. belladonnæ.
Ex. nucis vomicæ, aa gr. v. M.

Et. ft. in. pil. No. x.
Sig. One pill at bed time.

---

**Eczema of the Genitalia, Pruritus and Leucorrhœa.**

In cases of eczema, in which glyceroles and unguents have failed, the following formula has been successful:

℞

Chlorate of potassium, 30 grms.
Wine of opium, 50 grms.
Pure water, 1 quart.

Applied to the parts by linen compresses covered with oil silk. If there is much inflammation, precede this with warm hip baths and cataplasms sprinkled with powdered carbonate of lime. In obstinate pruritus, associated with leucorrhœa, a tablespoonful of mixture of equal parts of tincture of iodine and iodide of potassium, in a quart of warm tar water (tar water holding the iodine in solution) used daily, night and morning, removes the pruritus and ameliorates the leucorrhœa. In fetid leucorrhœa two or three tablespoonfuls (in a quart of warm water morning and evening, as an injection) of the following formula will be found useful:

℞

Chlorate of potassium, 12 grms.
Wine of opium, 10 grms.
Tar water, 300 grms.

Or,

℞

White vinegar (or wine), 300 grms.
Tinct. eucalyptus, 45 grms.
Acid salicylic, 1 grm.
Salicylate of soda, 20 grms.

One to five teaspoonfuls in a quart of warm water as an injection two or three times a day. —*Obstetric Gazette.*

**Angina Pectoris and Other Forms of Cardiac Pain.**

Balfour says that arsenic is indispensable in all forms of weak heart accompanied by pain. It is useful in all such cases, and in many cases it is quite successful in putting a stop to the pain. Several cases have occurred to me where arsenic alone has removed angina after a few weeks' treatment, not only temporarily but permanently. Its tendency to irritate the bowels in some patients may be overcome by the addition of opium or diminuation of the dose. One granule of Nativelle's digitalin, night and morning, with arsenic, strychnia and iron twice a day, after food, is a sort of model treatment in such cases. This treatment is often attended with the happiest results in those cases susceptible to improvement, which are by no means of infrequent occurrence.—*Louv. Med. News.*

---

**Orchitis.**

I know of no remedy that will allay the pain, and subdue the inflammation so effectually, as a tobacco and flaxseed meal poultice. The application was used by some New York surgeons long before it became much known, and Van Buren and Keyes have probably done more than any others to spread the knowledge of its use. A hot flaxseed meal poultice to which has been added previously about half a paper of fine cut chewing tobacco, should be applied fresh two or three times a day, until the swelling and pain subside. A piece of oil silk should be placed outside the poultice to prevent evaporation. This poultice gives relief quickly, and in the course of a few days the swelling is so reduced that an ordinary suspensory bandage may be worn with comfort.—*Philadelphia Medical Times.*

---

**Micrococci.**

The presence of Micrococci in the Blood of Malignant Measles, and its importance in treatment, is the title of an interesting and suggestive article in the *Medical Times*, by Dr. John M. Keating. From it we gather the following: "The observations were made during an epidemic in the Children's Asylum of the Philadelphia Hospital. Daily examination of the blood of each little patient showed the presence of micrococci in the malignant cases and their absence in those of mild type. Upon *post mortem* examination micrococci were found in the liquor sanguinis and the white blood corpuscles, and they were mobile. In the corpuscles they were seen in great numbers in active movement of a vibratory or whirling character, and they appeared to have devoured the white cells. Similar examinations in many cases gave similar results."—*Chicago Medical News.*

---

**Catarrhal Conditions.**

Dr. Goodwillie, of New York, recommends powders to be used by insufflation in catarrhal affections, and gives the following recipes for those he has found most useful:

No. 1.

℞

Benzoini, ʒ j.
Morph. muriat, grs. vj.
Bismuthi subnitrat, ℥ ss.
Potassii nitrat, ℥ ss. M

Valuable for its sedative action. To be used in hyperæmic conditions with pain. In the beginning of an attack of rhinitis, coat the mucous surface with it.

No. 2.

℞

Aluminis, ʒ j.

Acaciæ, ʒ iv.

Bismuthi subnitrat, ʒ iv.

Potassii nitrat, ʒ iv. M

Useful where a strong astringent is indicated.

In case of hæmorrhage from the nose, remove all the clot and immediately blow in this powder abundantly until the bleeding ceases.

No. 3.

℞

Iodoform, ʒ j.

Camphoræ, ʒ j.

Bismuthi subnitrat, ℥ ss.

Potassii nitrat, ℥ ss.

A good antiseptic.

To be used where the discharges are fœtid, or where ulceration is present or an excessive amount of granulations.

The camphor masks the odor of the iodoform.

These powders, when impalpable and with the therapeutic integrity of the drugs preserved, can be more effectually applied to the nasal passages than spray, and their good effect is certainly more prolonged.

For the general practitioner they are vastly more convenient than sprays.—*St. Louis Druggist.*

**Remedies for Headache.**

*Citrate of Caffeine, or Caffeine.*—Two grains every half hour, in capsules, if preferred, or triturated with milk sugar. It may cause sleeplessness with some people, if taken in the evening, but is preferable to guarana, as less liable to disturb the stomach.

*Chloride of ammonium*, 3 drachms; acetate of morphia, 1 grain; citrate of caffeine, 30 grains; aromatic spirit of ammonia, 1 drachm; elixir of guarana, 4 ounces; rose water, 4 ounces. Mix. A dessert-spoonful to be taken at intervals of ten to fifteen minutes. This is given on the authority of Dr. W. W. Carpenter, but is open to the objection of being polypharmacal—a shot-gun prescription, so to speak.

*Oxide of zinc*, 2 to 5 grains, thrice daily after meals. Give in pillular form.

*Nux vomica*, $\frac{1}{8}$ grain of the extract, after meals. If the case is one of chlorosis, add 1 grain of reduced iron, and $\frac{1}{2}$ grain of sulphate of quinine.

*Subcarbonate of bismuth*, 2 (?) grains (so W. H. Hammond says), after meals, when indigestion is a cause. The dose seems very small.

*Bromides* are serviceable when headache follows excitement, but is said to be likely to do harm rather than good when the nervous system is exhausted.

*Phosphorus*, in the form of dilute phosphoric acid, in dose of 30 drops, well diluted, thrice daily, and after eating, or in the form of *phosphide of zinc*, $\frac{1}{10}$ grain, thrice daily.

*Arsenic*, 5 drops of "Fowler's solution," or solution of arsenite of potassium, taken thrice daily, well diluted, and after eating. This will be found especially serviceable in the headache accompanying the menopause.

*Epsom salt* is said by Dr. T. Lauder Brunton to be serviceable, either in the form of a full dose, to secure brisk purgation, or small doses, taken twice daily, when frontal headache is associated with constipation. It may be noted, however, that saline purgatives are not as well borne, as a rule, by the people of this country as by those of Great Britain. The same authority recommends 10 drops of nitro-muriatic acid (dilute ?), in a wineglassful of water, when the bowels are already acting normally.

*Bicarbonate of sodium*, 10 grains in water, before meals. This is most serviceable when frontal headache is high up, at the margins of the hairy scalp, the nitro-muriatic acid being preferable to it when the headache is located at the eyebrows.

*Cannabis Indica*, 10 minims taken thrice daily, before the attack, is said to have proved remarkably curative of sick-headache in females when the disease seemed to be of hereditary character.

*Iris versicolor*, in minute doses, at short intervals, for sick headache accompanied with disturbances of vision like floating specks, or flashes of light, colored rays, etc.

*Hot water*, applied by means of a towel or flannel to the head. In the use of this remedy, it must be remembered that the water should be as hot as can well be borne.

*Iodide of Potassium*, 2 grains, dissolved in half a wineglassful of water, and sipped so that its consumption will consume about ten minutes, is said to be almost a specific in headache of a dull, heavy character, situated over the eyebrows, and accompanied by languor, chiliness, general discomfort, and distaste for food approaching to nausea.

*Oil of Peppermint.*—The Chinese oil may be used by wetting a needle with it, and drawing the point slowly across the forehead; a sense of coolness along the track of the needle follows, and in many cases the headache quickly disappears.

*Citrate of Caffeine.*—One grain, every half-hour, often produces most marked relief in migraine.

*Gelsemium.*—Three-minim doses of the tincture, every half-hour, will often relieve miraculously, neuralgias about the face or head, and leave no ill effects. This drug should be given with great caution, else is disturbance of vision caused, that may continue for weeks. Three-minim doses every half hour is too frequent.—ED. BISTORY.

*Guarana*—Fifteen-minim doses of the fluid extract, given every fifteen minutes, frequently relieves headaches, especially when they are periodical and not of malarial origin.

**Gout.**

Dr. Kendall writes to the *British Medical Journal* that he considers the iodide of lithia a most useful remedy in gout, and in many affections of a gouty nature.

**Sciatica.**

Dr. Pollak stated, at a recent meeting of the St. Louis Medico Chirurgical Society, that he had relieved himself of sciatica by an injection of cold water.—*St. Louis Courier of Medicine.*

